# Comparison between Resonance and Non-Resonance Type Piezoelectric Acoustic Absorbers

**DOI:** 10.3390/s20010047

**Published:** 2019-12-20

**Authors:** Joo Young Pyun, Young Hun Kim, Soo Won Kwon, Won Young Choi, Kwan Kyu Park

**Affiliations:** Department of Convergence Mechanical Engineering, Hanyang University, Seoul 04763, Korea; jooyoungpyun@hanyang.ac.kr (J.Y.P.); jason401@hanyang.ac.kr (Y.H.K.); rnjstndnjs@hanyang.ac.kr (S.W.K.); cwy1533@hanyang.ac.kr (W.Y.C.)

**Keywords:** resonance model, non-resonance model, piezoelectric material

## Abstract

In this study, piezoelectric acoustic absorbers employing two receivers and one transmitter with a feedback controller were evaluated. Based on the target and resonance frequencies of the system, resonance and non-resonance models were designed and fabricated. With a lateral size less than half the wavelength, the model had stacked structures of lossy acoustic windows, polyvinylidene difluoride, and lead zirconate titanate-5A. The structures of both models were identical, except that the resonance model had steel backing material to adjust the center frequency. Both models were analyzed in the frequency and time domains, and the effectiveness of the absorbers was compared at the target and off-target frequencies. Both models were fabricated and acoustically and electrically characterized. Their reflection reduction ratios were evaluated in the quasi-continuous-wave and time-transient modes.

## 1. Introduction

Piezoelectric transducers are used in various fields, such as nondestructive evaluation, image processing, acoustic signal detection, and energy harvesting [1,2,3,4]. Sound navigation and ranging (SONAR) is a technology for acoustic signal detection that can be used to detect objects under water. Stealth technology has been developed to prevent detection by such SONAR systems. Therefore, studies have focused on reducing the reflection of sound waves to prevent detection by SONAR systems. Wedge shaped structures or coatings have been used as passive sound absorbers to minimize underwater acoustic reflection. The coating is durable, but needs improvement. Therefore, minimizing detection in the low frequency range is challenging. In the low frequency range, the wavelength of the signal is several tens of centimeters. The thickness of the wedge shaped structure or coating should be at least several tens of centimeters to minimize acoustic reflection. Attaching a thick, passive sound absorbing material to a submarine hull increases the weight of the submarine and interferes with its propulsion. Sound can also be reduced using the wave scattering method. However, this technique only works under hydrostatic pressure [5,6]. Therefore, active sound absorbing materials have been developed to overcome the disadvantages of passive sound absorbing materials [7,8,9,10]. Lafluer et al. used piezorubber to realize an active sound absorbing material [11], whereas Howarth et al. used piezoelectric composites to reduce reflection. Although this material exhibited high attenuation performance at one frequency (5.4 kHz), it was not an active sound absorbing material composed of one structure. [12]. Chang et al. reduced sound reflection using two layers of a 1–3 piezoelectric composite. This resulted in an attenuation performance of 20 dB in the range of 6–10 kHz [13]. Accordingly, piezoelectric acoustic absorbers can be classified into resonance and non-resonance types based on previous studies. However, it is difficult to compare them due to the different approaches involved. Therefore, we believe that only resonance and non-resonance models with similar structures should be compared. In this study, resonance and non-resonance models suitable for the target frequency were designed via mathematical analysis. Based on the designs, the resonance and non-resonance models were fabricated and evaluated. The absorber was configured in the form of tiles, and the lateral size of the structure was equal to half of the wavelength. Our proposed resonance and non-resonance models were fabricated by stacking commercially available lead zirconate titanate (PZT). Various studies have previously investigated the stacking or direct fabrication of devices [14,15,16,17,18,19]. 

## 2. Materials and Methods

### 2.1. Concept

In the case of a piezoelectric material, vibration along the thickness direction is observed when an electrode is applied, and the sound spreads around both the front and rear sides of the piezoelectric material. The resonance frequency range of a piezoelectric material depends on its thickness. The thickness of a piezoelectric material and its resonance frequency are inversely proportional, i.e., the thinner the piezoelectric material, the higher is its resonance frequency and vice versa.

In this paper, we present a model that resonates in the low frequency region of the target frequency and a non-resonance model that deviates from the target frequency. The proposed resonance and non-resonance models incorporated a function that cancelled a certain portion of the incident sound waves. 

**Figure 1:**
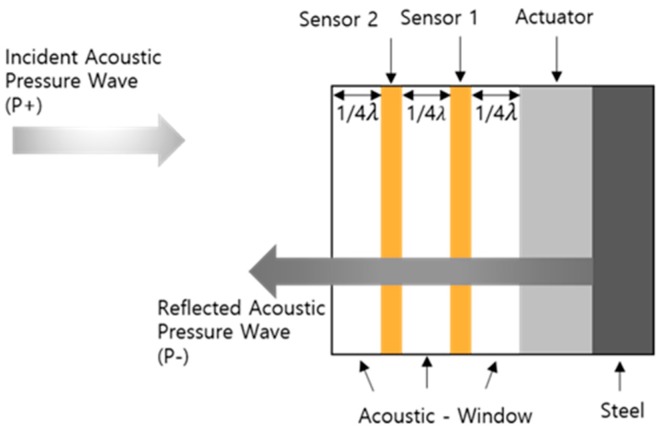
Incident and reflected waves.

The incident and reflected waves must be separated to cancel the incident sound wave. Two receiving sensors were required to separate the incident and reflected waves, as the signals were measured based on the overlap between these two waves. The incident ($P^{+}$) and reflected ($P^{–}$) acoustic pressures were the input and output of the system, respectively (Figure 1). We calculated the incident and reflected acoustic sensitivities based on the different receiving sensitivities of the two sensors obtained using the Krimholtz–Leedom–Matthaei (KLM) model. 

**Figure 2:**
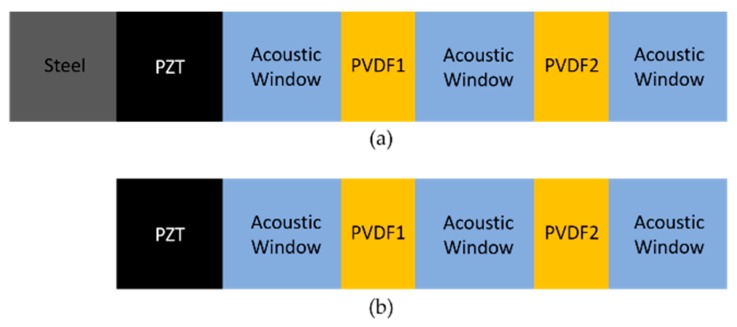
Concept behind the (**a**) resonance and (**b**) non-resonance models.

The proposed resonance and non-resonance models are shown in Figure 2. Generally, PZTs with low resonance frequencies are difficult to manufacture. Therefore, the frequency was reduced by attaching steel to the rear side of the PZT. The model with steel at the rear side of the PZT was the resonance model, whereas that without steel was the non-resonance model, as shown in Figure 2. Both models consisted of one transmitting sensor, two receiving sensors, and three acoustic windows. Polyvinylidene difluoride (PVDF) could be used as a receiving sensor to measure the sound pressure without disturbing the sound propagation. The transmitter used PZT-5A, which is insensitive to temperature changes and has excellent transmission capabilities. The resonance and non-resonance models presented herein were used underwater. Therefore, a physical gap was required between the two receiving sensors. The acoustic window material Rho-c was used because it is a lossy absorber with a physical gap and an impedance similar to that of water. 

### 2.2. Analytical Model

We adopted a mathematical analysis that required less time compared to the finite element method to analyze the resonance and non-resonance models composed of multiple layers.

**Figure 3:**
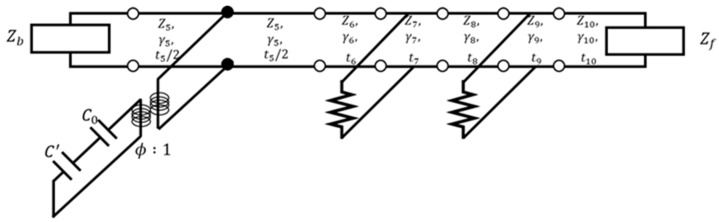
The Krimholtz–Leedom–Matthaei (KLM) model.

Some basic mathematical analysis methods have been developed for analyzing piezoelectric transducers [20,21,22], of which the KLM model is the simplest. In the KLM model, transducers are divided into two parts, namely their front and rear ends. In addition, matching layers can be added to the front and rear ends of the transducer. Therefore, the KLM equivalent circuit model can be used to analyze a multi-layer model. As shown in Figure 3, our model consisted of one transmitting sensor and two receiving sensors, with three piezoelectric materials and three matching layers. The equivalent models included capacitors, a transformer, piezoelectric materials, and matching layers.

(1) $$c_{0}=\;\frac{\varepsilon A_{1}}{d}$$

(2) c′=–c0kt2sinc(ωω0)

(3) $$\phi =\frac{k_{t}}{\sqrt{2f_{0}c_{0}Z_{c}}}sinc\left(f/2f_{0}\right)$$

Equations (1) and (2) were used to calculate the capacitances, and Equation (3) shows the transformer operation that converts the mechanical properties to acoustic properties. Here, $c_{0}$ is the static capacitance, ε is the permittivity, $A_{1}$ is the area, and d is the thickness of the piezoelectric material. $k_{t}$ is the electrical coupling constant. $f_{0}$ is the resonance frequency. sinc(x)=sin(πx)/πx, and $\omega_{0}=2\pi f_{0}$. The electrical ratio is represented by ϕ. The propagation constant γ can be calculated based on the velocity of sound and the quality factor.

(4) $$\;\gamma =j\frac{2\pi }{V}\left(1-\frac{j}{2Q}\right)$$

(5) $$M_{0}=\left[\begin{array}{cc} \cosh\gamma t & Z\sinh\gamma t\\ \frac{1}{Z}\sinh\gamma t & \cosh\gamma t \end{array}\right]$$

(6) $$M_{1}=\left[\begin{array}{c} 1\;\;\;\;\frac{–j}{\omega c^{\prime }}\\ 0\;\;\;\;\;\;1 \end{array}\right]$$

(7) $$M_{2}=\left[\begin{array}{cc} 1 & \frac{-j}{\omega c_{0}}\\ 0 & 1 \end{array}\right]$$

(8) M3=[∅001∅]

(9) M4=[101Zba1]

(10) $$M_{5}=\;\left[\begin{array}{cc} cosh\frac{\gamma_{5}t_{5}}{2} & Z_{5}sinh\frac{\gamma_{5}t_{5}}{2}\;\\ \frac{1}{Z_{5}}sinh\frac{\gamma_{5}t_{5}}{2} & cosh\frac{\gamma_{5}t_{5}}{2} \end{array}\right]$$

(11) $$M_{i}=\;\left[\begin{array}{cc} cosh\gamma_{i}t_{i} & Z_{i}sinh\gamma_{i}t_{i}\;\\ \frac{1}{Z_{i}}sinh\gamma_{i}t_{i} & cosh\gamma_{i}t_{i} \end{array}\right],\;i=6,7,\;\cdots ,10$$

Matrices $M_{1}$–$M_{3}$ represent the electrical parts of the piezoelectric transducer; $M_{4}–M_{5}$ represent the mechanical parts; $M_{7}$ and $M_{9}$ represent the receiving sensors; $M_{6},M_{8},$ and $M_{10\;}\mathrm{represent}$ the acoustic window matrices; $M_{1}$ and $M_{2}$ represent the capacitances. The values of $c_{0}$ and $c^{\prime }$ should be calculated to express the matrices $M_{1}$ and $M_{2}$. The permittivity ε, area *A1*, and thickness *d* of the piezoelectric material should be considered to obtain the value of $c_{0}$. The matrix $M_{3}$ represents the transformer. The transformer is transformed into an acoustic system when the capacitances exhibit electrical performance. $M_{4}$ and $M_{5}$ can be used to analyze the piezoelectric material. The piezoelectric matrix contains additional terms such as $Z_{5}$, $\gamma_{5}$, and $t_{5}$, which are the impedance, propagation constant, and thickness of the piezoelectric material, respectively. $M_{4}$ and $M_{5}$ represent the front and rear sides of the piezoelectric material, respectively. Therefore, the total thickness of the piezoelectric material should be substituted by half the actual thickness. $M_{5}$ and $M_{6}$ can be derived from the delay line, $M_{0}$.

(12) $$M_{total}=\;M_{1}M_{2}M_{3}M_{4}M_{5}M_{6}M_{7}M_{8}M_{9}M_{10}$$

The matrix $M_{total}$ can be constructed as shown in Equation (12). Subsequently, $M_{total}$ is employed to construct the matrices A–D as shown in Equations (13) to (16).

(13) $$A=\;M_{total\;}\left(1,1\right)$$

(14) $$B=\;M_{total\;}\left(1,2\right)$$

(15) $$C=\;M_{total\;}\left(2,1\right)$$

(16) $$D=\;M_{total\;}\left(2,2\right)$$

(17) $$Z_{e}=\;\frac{V}{I}=\;\frac{AZ_{F}+B}{CZ_{F}+D}$$

(18) $$v=\;\frac{–V}{AZ_{F}+B}$$

The electrical impedance of the transducer $Z_{e}$ can be calculated using Equation (17). The surface velocity of the transducer v can be calculated using Equation (18). Therefore, the transmit sensitivity of the transducer can be calculated using Equations (17) and (18). 

In the receiving model of the sensor, the matrix M is constructed in the same way as in the transmission model. The voltage V can be calculated based on the value of $M_{total}$, which is the product of the matrix of each property, the force applied to the transducer, and the value of the resistor.

(19) $$V=\;F_{s}\frac{R_{s}}{Z_{f}\left(CR_{s}+A\right)+DR_{s}+B}$$

The transmit characteristics, receiving characteristics, and input impedances of the resonance and non-resonance models were calculated using the KLM method. The frequency response characteristics were evaluated. The pole and zero within the frequency were specified. We developed and simulated a time transient model.

### 2.3. Control System

**Figure 4:**
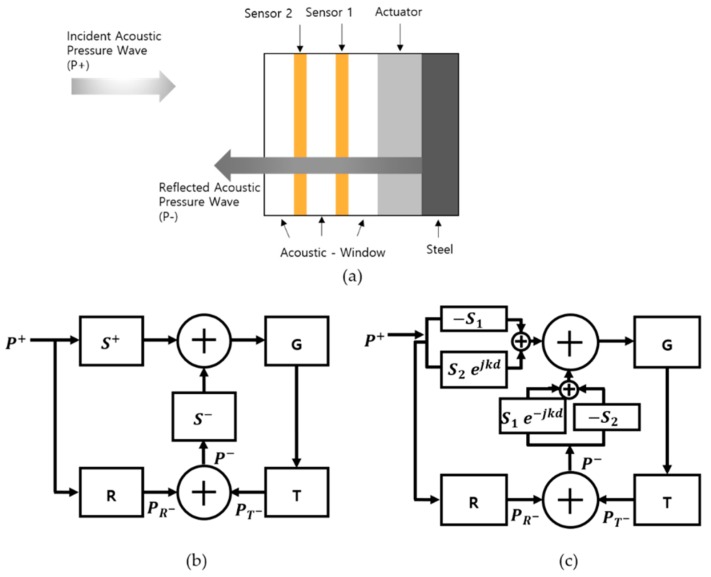
Block diagram of the feedback control (**a**) control system concept [12] (**b**) previous design (**c**) new design.

A basic active control system was built using the feedback controller design with two receiving sensors (Figure 4a). The previous design, shown in Figure 4b, was built based on the receiving sensitivity of the sensor (S), reflection sensitivity (R), and transmit sensitivity (T). The incident acoustic pressure ($P^{+}$) and total reflected acoustic pressure ($P^{–}$) were the input and output of the system, respectively. The incident ($S^{+}$) and reflected ($S^{–}$) acoustic sensitivities were calculated based on the sensitivity of the sensor (S) and the thickness of the acoustic window (d) using Equations (20) and (21), as shown below.

(20) $$S^{+}=j2Ssin\left(kd\right)e^{jkd}$$

(21) $$S^{-}=-j2Ssin\left(kd\right)$$

Unlike in the previous design (Figure 4c), we calculated the new $S^{+}$ and $S^{–}$ values from the different receiving sensitivities of the two sensors calculated using the KLM model. $S^{+}$ was calculated using Equation (22), as there were phase differences at the two sensors. $S^{–}$ was calculated using Equation (23).

(22) $$S^{+}=S_{B}e^{jkd}-S_{A}$$

(23) $$S^{-}=-S^{+}e^{–jkd}$$

The gain G was maintained constant, with its optimal value obtained after designing the model for the target frequency [12].

### 2.4. Analysis Results

The transmit and receiving sensitivities of the models were analyzed in the frequency domain. All the dimensions were normalized with respect to the wavelength of the target frequency, λ, and the period of the target frequency, T, to evaluate the performance in the normalized dimension. The dimensions of the resonance and non-resonance models were the same as those of the fabricated structure discussed in the previous section. Both models had the same cross-sectional area of 0.36λ × 0.36λ. The heights of the resonance and non-resonance models were 2.7λ and 1.2λ, respectively. The parameters used in our simulations are listed in Table 1.

**Figure 5:**
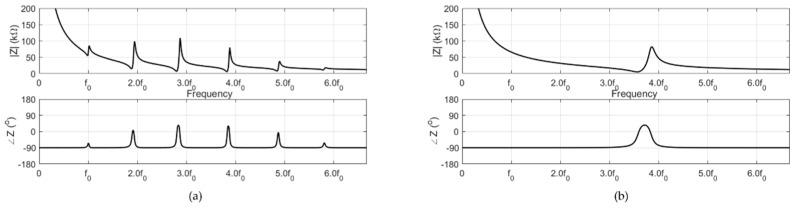
Simulation of the input impedances of the (**a**) resonance and (**b**) non-resonance models.

**Figure 6:**
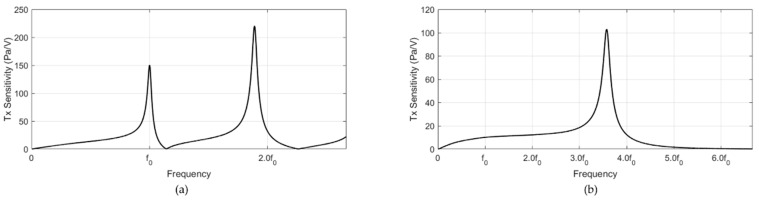
Simulation of the transmit sensitivities of the (**a**) resonance and (**b**) non-resonance models.

Each model had one transmit element (PZT) and two receiving elements (PVDFs). The input impedances of the transmit elements of both models were calculated as shown in Figure 5 to evaluate the frequency response of the system. The input impedance confirmed that the electrical resonance of the resonance model was at the target frequency $f_{0}$. In addition, the input impedances of both models were affected by the steel blocks, which reduced the resonance frequency of the PZT from $3.6f_{0}$ to $f_{0}$ at the cost of the system bandwidth. Notably, both models employed the same piezoelectric transmitter and thus had identical off-resonance characteristics such as capacitance. The output pressure of the system for a small voltage input can be calculated as shown in Figure 6. As the resonance model had a high quality factor around the resonance, it had a higher transmit sensitivity (150 Pa/V at $f_{0}$) than that of the non-resonance model (102 Pa/V at $3.6f_{0}$). The transmit sensitivity results demonstrated the benefit of the resonance structure at the target frequency, as this structure had 15 times higher transmit sensitivity than the non-resonance structure (10 Pa/V at $f_{0}$).

**Figure 7:**
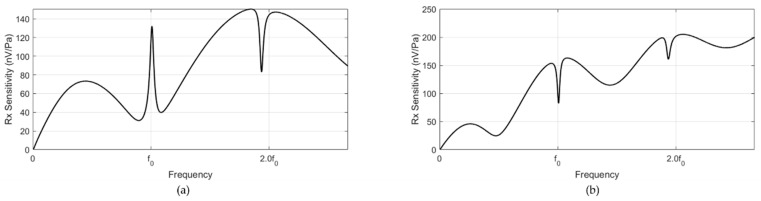
Receiving sensitivities of the (**a**) PVDF1 and (**b**) PVDF2 resonance models.

**Figure 8:**
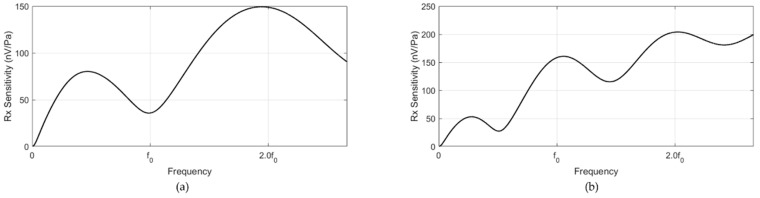
Receiving sensitivities of the (**a**) PVDF1 and (**b**) PVDF2 non-resonance models.

The receiving sensitivities of the two receivers exhibited an excellent broad frequency response. (Figure 7 and Figure 8) The thickness of the PVDF film was 0.002λ; thus, its resonance frequency was significantly higher than the frequency range of interest. Due to the lossy factor of the acoustic window, the receiver close to the transmitter (PVDF1) had a lower receiving sensitivity than that of the outer receiver (PVDF2) (Figure 7 and Figure 8). Overall, no difference was observed between the receiving sensitivities of the resonance and non-resonance models, except that the resonance model presented dramatic changes in the receiving sensitivity around the resonance frequencies of the structure. 

**Figure 9:**
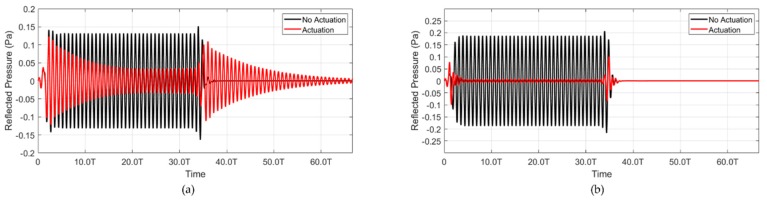
Simulations showing the cancellation results. (**a**) Resonance and (**b**) non-resonance model.

The frequency response characteristics of both models were converted into a time transient model, which was attached to a feedback controller, and time transient analysis was performed. The gain of the controller was pre-calculated based on the receiving and transmit sensitivities of the model in the frequency response. A tone-burst sine waveform was applied to both models, and the reflected sound was calculated. As the system was considered linear, 1 Pa amplitude, 20 cycles at $f_{0}$ sound wave were applied to the model. The typical response of the reflected sound wave from the model is shown in Figure 9. When the controller was switched off, 20% of the sound was reflected because of the lossy acoustic windows used in the model. The reflected sound level dramatically reduced with the actuation of the transmitter. In the continuous wave (CW) condition, the reflected wave was reduced to 0.7% of the input pressure. However, the reflected sound was approximately 10% (−20 dB) in the time transient region of the burst due to the low fractional bandwidth (FBW) of the system. Table 2 shows the reflection control simulation results for the resonance and non-resonance model in the CW and transient regions. 

The models were evaluated at the off-target frequency. The input frequency was changed from $0.8f_{0}$ to $1.2f_{0}$, and the reflection ratio of both models was calculated. The non-resonance model exhibited a better performance by 4 dB as compared with the resonance model at frequencies below $f_{0}$. Interestingly, both models showed a similar cancellation performance above $f_{0}$. Overall, the non-resonance model presented a broader cancellation response compared with the resonance model. 

**Figure 10:**
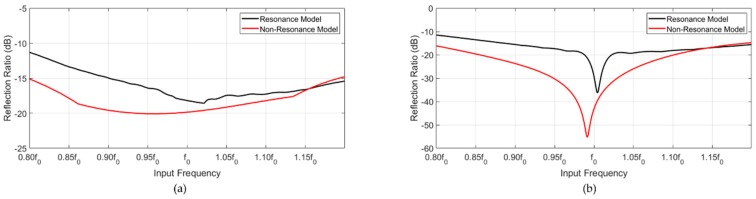
Reflection ratios of the resonance and non-resonance models. (**a**) Transient region and (**b**) CW region.

As shown in Figure 10, the reflection ratio in the time transient region was as follows. The resonance model was attenuated by −18 dB at $f_{0}$, and the non-resonance model was attenuated by −20 dB. In the CW region, the resonance model was attenuated by −30 dB at $f_{0}$, and the non-resonance model was attenuated by −43 dB at $f_{0}$.

**Figure 11:**
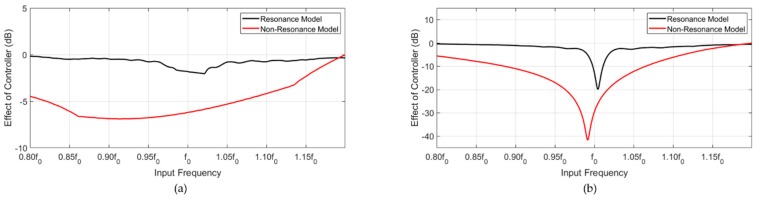
Effect of the controller of the resonance and non-resonance models. (**a**) Transient region and (**b**) CW region.

Figure 11 shows the effect of the controller. In the time transient region, the resonance model was attenuated by −2 dB at $f_{0}$. The non-resonance model was attenuated by −6 dB at $f_{0}$. In the CW region, the resonance model was attenuated by −15 dB at $f_{0}$, and the non-resonance model was attenuated by −29 dB at $f_{0}$. In the case of the designed controller, the results for the two models showed that the effect was drastically reduced at the off-resonance frequency. However, the deviation from the peak in the non-resonance model was less attenuated than that in the resonance model. 

### 2.5. Fabrication

Our aim was to develop resonance and non-resonance models suitable for operation in the low frequency range. 

**Figure 12:**
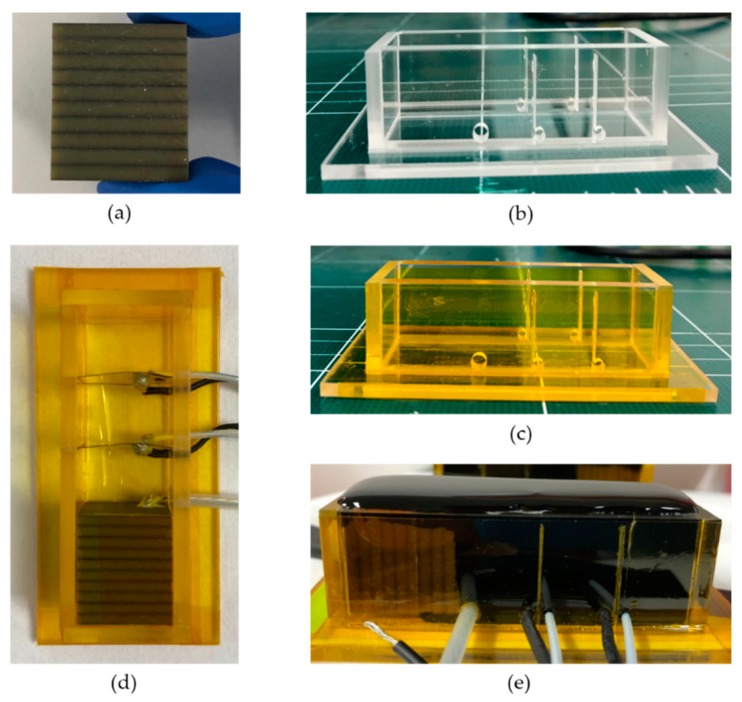
Fabrication process: (**a**) Stack PZT, (**b**) acrylic mold, (**c**) mold assembly and taping, (**d**) transmit and receiving sensor connection, and (**e**) curing acoustic window.

The resonance and non-resonance models required a thick PZT layer in order to be suitable for the low frequency range. PZT is a piezoelectric material; hence, it should be polled to vibrate due to the voltage difference. However, it is not feasible to obtain PZT with a polling of tens of centimeters. Therefore, we used a thick PZT layer wherever possible and attached a backing material to the rear side of the PZT to increase the output performance [23]. Thus, we present a model that achieved the target frequency. The resonance and non-resonance models were composed of ten layers of commercially available PZT-5A, excluding the low frequency transducers. As shown in Figure 12a, low frequency piezoelectric material based transducers were fabricated. Accordingly, 2 mm thick PZT-5A (T180-A4N0-2929, Piezo.com, Woburn, MA, USA) was stacked to implement the transducer for low frequency operation. As the resonance and non-resonance models would be used underwater, instead of PZT-5H, which has excellent output performance, PZT-5A was employed due to its insensitivity to temperature changes. When stacking PZT-5A, EPOTEK-301 (EPOTEK 301, Epoxy technology, Billerica, MA, USA), which has a low viscosity and excellent adhesion, was used (Figure 12a). EPOTEK-301 consists of Parts a and b, which were mixed using a weight ratio of 4:1 and stirred for 5 min. We used Parts a and b after they were sufficiently shaken. Subsequently, the bubbles formed were removed using a vacuum pump. The interior of the vacuum pump was maintained at 100 kPa for 15 min. The degassed EPOTEK-301 was thinly applied on top of PZT-5A. If a large amount of EPOTEK-301 were applied, it might be difficult to establish a connection between the piezoelectric elements due to the thickness of the epoxy. Subsequently, the epoxy spilled on the PZT side was wiped off using a wiper, as the vibration mode of the PZT may change if the epoxy flowing down the side of the PZT is not removed. Each corner of PZT-5A was adjusted using a pair of tweezers and a slide glass 30 min after the application of EPOTEK-301.

If the cured PZT was not subjected to any slipping caused by the epoxy, a weight of 100 g was added and then cured at 65 °C for 2 h. Whenever the PZT was stacked, the same operation described above was performed, and the electrical impedance was measured. We verified whether the resonance frequency of the device decreased when the devices were stacked. Furthermore, when laminating the PZT-5A, it was ensured that each side faced upward. PZT was stacked in one direction using EPOTEK-301. The sides of the device were wrapped with Kapton tape when the PZT-5A was stacked up to ten layers. 

Ten layers were constructed with PZT, and subsequently, a mold was built. A 3 mm thick acrylic plate was incorporated on each side of the mold using a laser cutter (Figure 12b). When each side was completed, it was assembled to complete the mold. The appearance of the finished mold was fixed with Kapton tape. The prefabricated frame can be conveniently removed later. When the mold was developed as one piece, as opposed to assembling several parts, it was prefabricated because the sensor was damaged when the mold was removed. In this study, we present a horizontal mold, because when the layers were stacked vertically, the acoustic window hardened and caused shrinkage, resulting in a variable height. After the mold was completed, its interior was cleaned with an acetone swab to remove the powder from laser cutting (Figure 12c).

Subsequently, PZT and PVDF were placed inside the mold, and the wires were connected (Figure 12d). The transmitting sensor (PZT-5A) was placed at the bottom of the mold and was fixed using double sided tape. The receiving sensors (PVDF) were designed to fit on both sides of the mold, requiring no additional attachments. The electrode parts of the transmitter and receiver were hardened over one day at room temperature after connecting the wires with the conductive epoxy. Then, the two receiving sensors (PVDF) were grounded. The ground of the stacked sensor PZT-5A, the transmitter, was connected after the mold was completely cured. Conductive epoxy consisting of Parts A and B should be mixed for 1 min in a ratio of 1:1. It is recommended that the conductive epoxy be mixed on the box tape, as it penetrates the paper. Hot plates can be used to cure the conductive epoxy faster; however, this method is not recommended. The hardening on the hot plate showed that the receiving sensor was sensitive to heat, resulting in a deformed appearance. Therefore, the method of curing at room temperature was selected. In this case, each wire used a coaxial cable, and the wires were attached to the edge of the sensor to ensure that the sound waves of the sensor propagated without interference. The coaxial cable was thicker than a standard wire and was fixed by bending the wire with a pair of tweezers. This ensured that it did not float from the sensor and was attached to the conductive epoxy. We used a large amount of conductive epoxy and attempted to fix the wire only based on the weight of the conductive epoxy. However, this method did not succeed. Therefore, the wire height was adjusted accurately before applying the conductive epoxy. After the conductive epoxy was cured, a multimeter was used to check whether the electrodes were connected. 

The acoustic window (Rho-c) existing between the transmitter and the receiver was prepared. The acoustic window (Aptflex-F21, Precision Acoustics, London, UK) consisted of Parts A and B, which had to be shaken sufficiently before they could be used. Even if the weight ratio was adjusted, these parts did not perform as well as the original material of the acoustic window if not sufficiently shaken, and the acoustic window was gray without being black. Parts A and B were placed in a disposable plastic cup, with the weight ratio set to 3.35:1, and stirred for 5 min using wooden chopsticks. This stirring generated a chemical reaction that produced heat. Even if the parts were mixed for 5 min, the mixing did not generate heat, and the mixture was not cured even after the curing time, if the quantity was insufficient. Therefore, it is recommended that the mixture be stirred as fast as it is mixed. Subsequently, the bubbles generated while mixing were discharged with a vacuum pump outward. At this time, the vacuum pump was maintained at 100 kPa for 15 min. While the bubbles escaped, the interior of the mold was cleaned with acetone, using a cotton swab. The interior of the mold was wiped a few times. Acetone acted as a mold release. The acoustic window had to be prepared in less than 20 min, because after 20 min, it would harden inside the plastic container and become unusable. The acoustic window was then slowly poured into the mold. If the acoustic window exceeded the height of the mold, its height could be adjusted using a slide glass. The height was immediately flattened/reduced after pouring the acoustic window into the mold. However, if this process was delayed by a few minutes, the acoustic window would stick to the slide glass and exit the mold. Following the planarization operation, the acoustic window inside the mold exhibited several large and small bubbles even after bubble removal. The bubbles were popped using a toothpick. Once popped, bubbles were still generated, which had to be popped several times. A bubble in the acoustic window was popped using a toothpick, and when the acoustic window was completely attached to the toothpick, it was rolled up into a circular shape using the toothpick. The flattening process was then repeated. The acoustic window was left to cure for 1 to 2 days at room temperature, without covering the mold. Once the acoustic windows had cured, the mold was removed using a knife. Considerable care was exercised during this process to avoid damaging the sensor. Care was also taken when removing the lowest part of the horizontal mold. The mold was carefully removed, particularly where the PVDF was inserted. The mold in the PVDF region had to be removed faster than in the other parts to avoid splitting the PVDF into two (Figure 12e).

Once the upper part of the piezoelectric based resonance model was constructed, the ground was connected to the bottom of the stacked piezoelectric element via the conductive epoxy. Subsequently, the section connecting the acoustic window and electric wire was coated with epoxy resin for 5 min. This process was repeated two more times. Finally, we attached steel to the rear end of the PZT-5A stacked device using EPOTEK 301 to fabricate the resonance model further (Figure 12e). 

One hour after fixing, a weight of 200 g was added. The ground of the resonance model was connected to the steel using a conductive epoxy, as soldering was not effective due to the lubricant used in the steel processing. When the conductive epoxy was cured, the epoxy resin was applied to the conductive part for 5 min. This was necessary to fix and coat the ground. The resonance and non-resonance models were fabricated using the aforementioned processes.

### 2.6. Experimental Setup 

**Figure 13:**
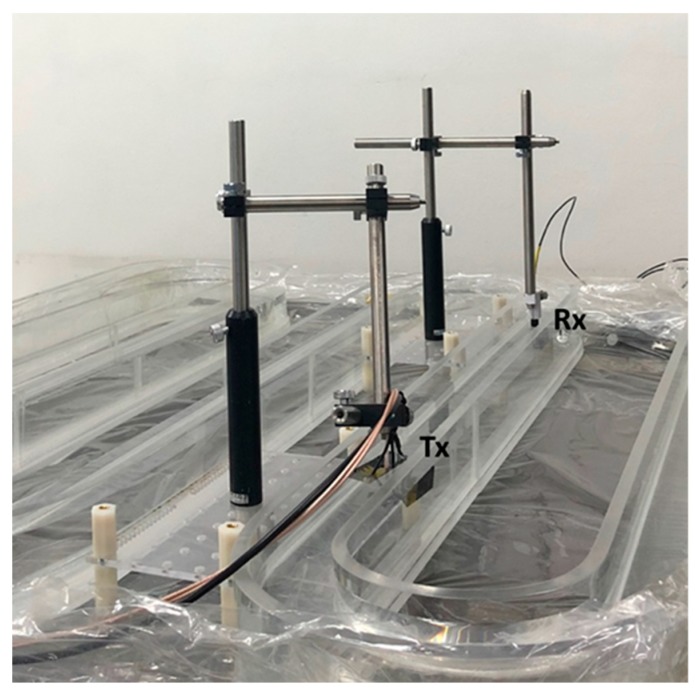
Evaluation of the transmission characteristics of the resonance and non-resonance models.

We measured the transmission characteristics of the resonance and non-resonance models in a snake shaped tank (Figure 13), which was composed of 10 mm thick acrylic and had a total length of 5.8 m. When measuring the transmission characteristics of a device in a tank of length 1 m, reflection occurred at the end surface of the tank, thus making it difficult to determine the transmission characteristics accurately. Therefore, the measurements were performed in a tank of a length of 5.8 m to minimize the reflection from the end surface of the tank. As shown in Figure 13, the experiment was conducted by filling the tank with water. The resonance and non-resonance models were located at the center of the tank and excited at 30 Vpp with ten cycle sine waves obtained using a function generator and amplifier. A low frequency hydrophone was placed at a distance of 600 mm from the resonance and non-resonance models. Low frequency hydrophones were used to measure the desired frequency range to evaluate the output performances of the resonance and non-resonance models.

**Figure 14:**
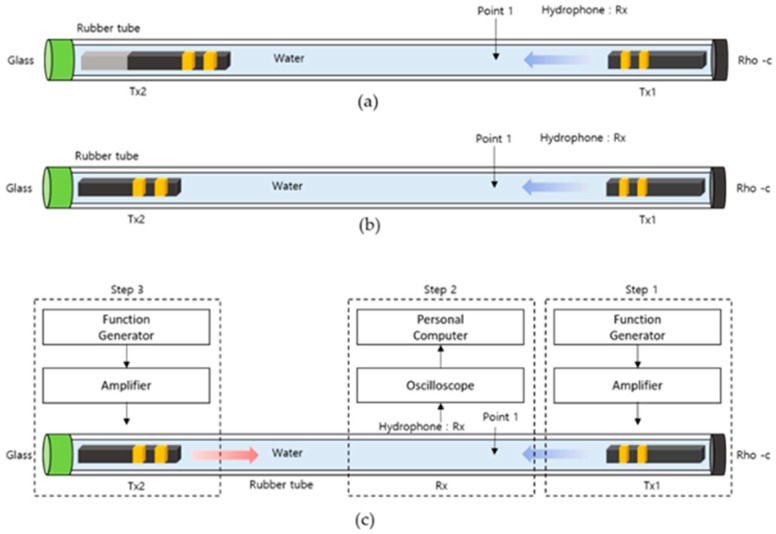
Experimental setup showing the cancellation in the resonance and non-resonance models. (**a**) experiment setup of resonance model (**b**) non-resonance model (**c**) experiment step.

The experimental setup used for the cancellation experiment is shown in Figure 14. As shown in Figure 14a, a rubber tube with inner and outer diameters of 35 mm and 45 mm, respectively, and a total length of 4.8 m was used as a water tank. The left side of the rubber tube was molded with glass and the right side with rubber. The hydrophone was located at a distance of 1 m from the right end of the rubber tube. Stacked PZT based devices for transmission were located at both ends of the rubber tube. 

As shown in Figure 14a,b, the resonance and non-resonance models were evaluated. As shown in Figure 14c (Step 1), a function generator and amplifier were used to activate the device at the right end section. At 70 Vpp, a sine wave with 30 cycles was applied to the right end of the device, and the signal reflected from the glass located on the left side of the rubber tube was measured using a hydrophone. After measuring the signal from the right side, the function generator connected to the right sensor was switched off. In Step 2 (Figure 14c), a function generator and amplifier were used to activate the resonance and non-resonance models located at the left end section. At 70 Vpp, a sine wave with 30 cycles was applied to the non-resonance model. The signal obtained at this time was measured using a hydrophone. After verifying the sensor measurement results on the left and right sides, the amplitude and delay time to be applied to the resonance and non-resonance models were determined. In Step 3 (Figure 14c), the amplitude and delay time were modified and measured again. This process was repeatedly performed, as the appropriate amplitude and delay time would increase the cancellation effect. Subsequently, two function generators and amplifiers were connected to each device to resonate with the two devices simultaneously. Finally, we verified whether the desired area had been cancelled.

## 3. Results 

### 3.1. Experimental Results

**Figure 15:**
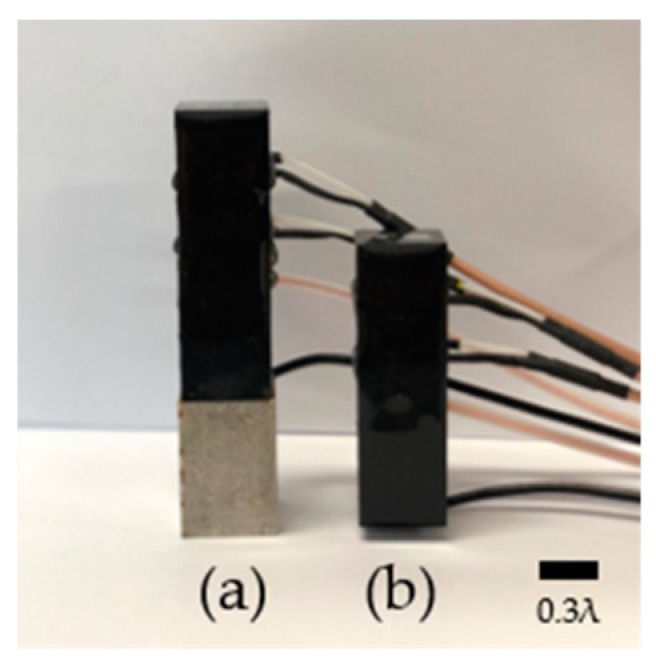
Fabricated (**a**) resonance and (**b**) non-resonance models.

The resonance and non-resonance models were fabricated with the same size and device. The resonance model shown in Figure 15 had a width, length, and height of 0.36λ, 0.36λ, and 1.8λ, respectively. The non-resonance model shown in Figure 15b had a width, length, and height of 0.36λ, 0.36λ, and 1.2λ, respectively. The wavelength was set based on the specific frequency and the velocity of sound in water. 

**Figure 16:**
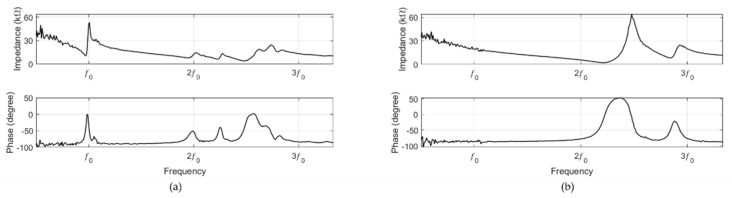
Input impedance of the (**a**) resonance and (**b**) non-resonance models.

The input impedance was measured after the fabrication of the resonance and non-resonance models. The impedance measurements were performed in air, and the measurement frequency range was $0.3f_{0}$ to $3.3f_{0}$. In Figure 16a,b, the resonance frequency can be observed at $0.97f_{0}$ and $2.17f_{0}$, respectively.

**Figure 17:**
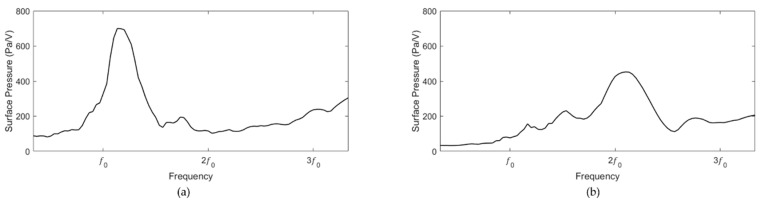
Transmit sensitivities of the (**a**) resonance and (**b**) non-resonance models.

The transmit characteristics of the resonance and non-resonance models were measured inside the snake shaped tank containing water. The measurement frequency range was $0.3f_{0}$ to $3.3f_{0}$. As shown in Figure 17a, the resonance model exhibited a performance of 699 Pa/V at $1.13f_{0}$. As shown in Figure 17b, the non-resonance model exhibited a performance of 452 Pa/V at $2.1f_{0}$. The resonance model demonstrated transmit characteristics that were 1.55 higher than those of the non-resonance model.

**Figure 18:**
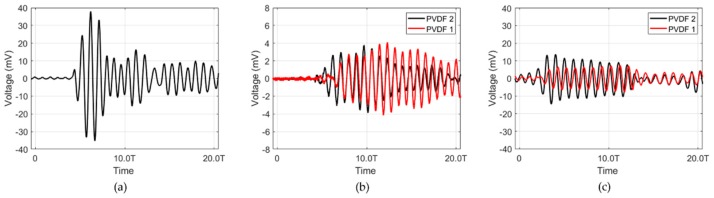
Received signal from the (**a**) hydrophone, (**b**) PVDF1 and PVDF2 resonance models, and (**c**) PVDF1 and PVDF2 non-resonance models.

Figure 18 shows the signal measured by the receiving sensors of the hydrophone and the resonance and non-resonance models when the external sound waves were transmitted to the transducer. When the external transducer was excited at 30 Vpp, the signal was received by the hydrophone, PVDF1, and PVDF2 inserted inside the resonance and non-resonance models. The signal received at the hydrophone, as shown in Figure 18a, was used to verify our resonance and non-resonance models. Figure 18b,c shows a scan of the received signals of the resonance and non-resonance models. Figure 18c shows that the signals of the receiving sensors differed by 1/4 of the wavelength. However, in the case of the resonance model, it was difficult to observe the 1/4 wavelength difference in the receiving sensor signals

**Figure 19:**
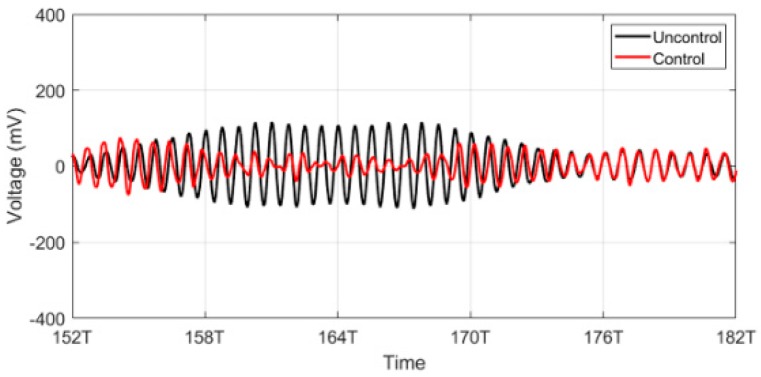
Cancellation.

We conducted cancellation experiments (Figure 19) on the resonance and non-resonance models. Table 3 shows the reflection control results for the resonance and non-resonance model in the CW and transient regions.

## 4. Discussion

This paper aimed to present the advantages and disadvantages of acoustic absorbers having the same parameters when operating in resonance or non-resonance modes. As described above, an absorber in resonance mode had a high transmit sensitivity relative to its resonance frequency. Thus, when a high output sound was incident, high cancellation may be demonstrated. When low pressure was applied, such as SONAR sound waves transmitted from a long distance, the non-resonance type showed excellent overall performance. In the case of the resonance type, the degrading performance was negligible except over the range of a small proportion of the resonance frequency due to the low FBW. The non-resonance type had a broader frequency response, except that it had a limited transmit sensitivity. Even in the non-resonance type, the FBW at the resonance frequency showed a narrow characteristic. The operating frequency was off-the resonance, which generally indicates a wideband output characteristic.

Two sensor approaches to separating input and output sounds are miniaturized methods widely used for acoustic characterization. Although they are mathematically concise and the design of the controller is easy, they have several disadvantages. First, in the two-sensor design, a 1/4 wavelength distance between sensors is required; thus, low frequency structure applications have thick layers. Furthermore, despite the wide FBW shown in Figure 17, this design only worked well around the designed target frequency. This paper presented a relatively simple control method for comparing the two types of absorbers. In addition, a modern cancellation method, such as the FxLMS (filtered – x LMS) algorithm, which has high accuracy, is expected to demonstrate excellent cancellation performance. Thus, it can be assumed that the non-resonance type will show excellent performance. We manufactured the resonance and non-resonance models inside our laboratory, without outsourcing to other companies. Therefore, even when the piezoelectric elements were stacked, the alignment was not even. Thus, it appeared that the piezoelectric element was affected during vibration. 

## 5. Conclusions

In this study, we analyzed and fabricated two different types of piezoelectric absorbers. The absorbers consisted of two receiving sensors composed of PVDF films and one transmitter made of PZT-5A. Both models were analytically analyzed in the frequency and time domains. With the conventional PZT and PVDF structures, the absorbers achieved a reflection ratio of greater than −17 dB in the CW condition and −4 dB in the time transient region. The resonance absorbers had high sensitivities around the resonance, albeit at the expense of the bandwidth. The non-resonance absorber exhibited a better frequency response; however, it required a high transmit voltage. The reflected sound wave was mainly observed in the time transient region when using a conventional feedback controller. Consequently, the resonance structure had a higher reflection ratio due to the narrow frequency response. This work showed that the fractional bandwidth of the system had an important role in feedback based piezoelectric absorbers. 

## Figures and Tables

**Table 1:** Simulation parameters.

	Resonance (Thickness: λ)	Non-Resonance Model (Thickness: λ)
Acoustic Window	0.27	0.27
PVDF	0.002	0.002
Acoustic Window	0.27	0.27
PVDF	0.002	0.002
Acoustic Window	0.27	0.27
PZT	0.4	0.4
Steel	1.52	-

**Table 2:** Reflection control of the resonance and non-resonance models. CW, continuous wave.

	Resonance	Non-Resonance Model
Reflection uncontrol (Pa)	0.15	0.2
Reflection control (Pa): transient region	0.12	0.1
Effect of actuation (dB)	−1.9	−6
Reflection control (Pa): CW region	0.03	0.007
Effect of actuation (dB)	−14	−29

**Table 3:** Reflection control of the resonance and non-resonance models.

	Resonance	Non-Resonance Model
Reflection uncontrol (mV)	152	116
Reflection control (mV): transient region	144	76
Effect of actuation (dB)	−0.5	−4
Reflection control (mV): CW region	40	16
Effect of actuation (dB)	−12	−17
